# Evaluating pain in non-verbal critical care patients: a narrative review of the critical care pain observation tool and Its clinical applications

**DOI:** 10.3389/fpain.2024.1481085

**Published:** 2024-10-15

**Authors:** Abebe Dilie Afenigus

**Affiliations:** Department of Nursing, College of Medicine and Health Sciences, Debre Markos University, Debre Markos, Ethiopia

**Keywords:** pain evaluation, non-verbal patient, critical care, critical care pain observation tool, clinical applications

## Abstract

**Background:**

Assessing pain in critically ill patients who cannot communicate verbally poses significant challenges. Traditional self-report measures are ineffective for these patients, making the need for reliable observational tools crucial.

**Objective:**

To evaluate the effectiveness, reliability, and clinical applicability of the Critical Care Pain Observation Tool (CPOT) in various intensive care unit (ICU) settings and to explore potential innovations for improving its use and integration into clinical practice.

**Methods:**

A narrative review evaluated the Critical Care Pain Observation Tool (CPOT) for non-communicative ICU patients, comparing it to the Behavioral Pain Scale (BPS) and the FLACC scale. The review assessed CPOT's effectiveness across different ICU settings, identified limitations and challenges, and explored potential enhancements such as electronic scoring, additional physiological indicators, and improved training protocols.

**Results:**

The CPOT has been validated as an effective pain assessment tool for non-verbal ICU patients. It evaluates pain through facial expressions, body movements, muscle tension, and ventilator compliance. The CPOT shows superior sensitivity at 76.5% compared to 62.7% for the BPS and offers a more comprehensive assessment of pain indicators like muscle tension and ventilator compliance than the FLACC scale. Despite its strengths, the CPOT has limitations, including inter-rater variability and challenges in certain patient populations. Barriers to implementation include resource constraints and the need for extensive training.

**Conclusion:**

The Critical Care Pain Observation Tool (CPOT) is a highly effective instrument for assessing pain in non-verbal ICU patients, demonstrating superior accuracy and reliability compared to other tools like the Behavioral Pain Scale (BPS) and FLACC scale. Its detailed approach, covering facial expressions, body movements, muscle tension, and ventilator compliance, offers a detailed measure of pain. However, challenges such as inter-rater variability and limitations in specific patient populations highlight the need for ongoing refinement and research.

## Introduction

Accurate pain assessment in critically ill patients, particularly those who are non-verbal, is significant challenge in critical care settings (1). These patients often lack the ability to communicate their discomfort verbally, making effective pain management critical for their overall care and recovery. Inadequate pain assessment can lead to unrecognized suffering, increased stress responses, and prolonged recovery times, ultimately impacting patient outcomes and quality of life (2).

Traditional self-report pain scales, which rely on patient communication, are not applicable for these non-verbal individuals, necessitating the development of reliable observational tools (3). The Critical Care Pain Observation Tool (CPOT) was primarily designed for use in ICU patients who are unable to self-report their pain, particularly those on mechanical ventilation. However, it can also be applied to other non-verbal ICU patients who may not be able to communicate their pain effectively, regardless of whether they are on mechanical ventilation. The tool assesses pain through observable behaviors and physiological indicators, making it versatile for various critically ill patients (2, 4).

CPOT was developed to improve pain assessment accuracy in patients who cannot self-report their discomfort, focusing on four key indicators: facial expression, body movements, muscle tension, and ventilator compliance (5, 6). The tool's development was driven by the need for a more comprehensive approach to pain evaluation in critically ill patients, particularly those who are intubated or deeply sedated (7). Validation studies have demonstrated that CPOT effectively identifies pain with high sensitivity and specificity, making it a valuable asset in critical care environments (6).

Despite its advantages, the application of CPOT in clinical practice is not without challenges. Issues such as inter-rater variability and the tool's performance in specific patient populations, including those with severe neurological impairments, have been noted (8, 9). Moreover, barriers to effective implementation, such as resource constraints and the need for extensive training, can impact the consistent use of CPOT across different ICU settings (10). This narrative review aims to evaluate CPOT's effectiveness, explore its clinical applications, and identify areas for improvement to enhance pain management in non-verbal critical care patients.

### Objectives

The primary objective of this review is to identify and evaluate the existing literature on the Critical Care Pain Observation Tool (CPOT) and its applications in assessing pain among non-verbal patients within critical care settings.

## Methods

### Inclusion and exclusion criteria

This review included only peer-reviewed studies such as randomized controlled trials, observational studies, cohort studies, and systematic reviews that focus on the effectiveness of the Critical Care Pain Observation Tool (CPOT). Eligible studies must involve critically ill patients in intensive care settings who are unable to communicate verbally due to sedation, intubation, or severe neurological impairments resulting from various diseases process. Only studies published in English up to the year 2023 that have obtained ethical approval from relevant institutional review boards were considered. While recent studies are often prioritized for relevance, older studies can provide valuable foundational insights, historical context, or demonstrate the evolution of concepts in the field.

Conversely, the review excluded anecdotal reports, case studies, opinion pieces, editorials, and non-peer-reviewed articles. Studies focusing on patients who can communicate verbally or who are not critically ill, such as those in outpatient settings, were also excluded. Articles that do not directly assess CPOT's effectiveness or lacks relevant pain assessment outcomes were filtered out. Additionally, duplicate publications presenting overlapping data were excluded, as were studies with insufficient sample sizes or incomplete data regarding CPOT's application, reliability, or validity. Lastly, studies primarily focused on other pain assessment tools was not be considered. Similarly, studies lacking full texts and duplicate articles were also excluded. Based on these criteria, a total of 200 articles were retrieved, of which only 25 were included in the review, while 175 articles were excluded according to the specified parameters.

### Search databases

To conduct a comprehensive search on a wide range of peer-reviewed literatures, several key databases were utilized, including PubMed, MEDLINE, CINAHL, Web of Science, Cochrane Library, PsycINFO and Google Scholar.

### Search terms

A strategic combination of search terms was employed using Boolean operators to refine the search results. The primary search terms were included “Critical Care Pain Observation Tool,” “CPOT,” “pain assessment,” “non-verbal patients,” “pain in critically ill patients,” “behavioral pain assessment,” and “pain evaluation ICU.” This combination is designed to yield comprehensive results focusing on the tool's application in the target population.

### Search strategy

The search strategy involved combining search terms with Boolean operators (AND, OR) to ensure relevant literature is captured. For example, the search was structured as follows: (“Critical Care Pain Observation Tool” OR “CPOT”) AND (“non-verbal” OR “non-communicating”) AND (“pain assessment” OR “pain evaluation”). Filters was applied to restrict study types. Additionally, reference lists from included studies were reviewed to identify any further relevant articles that may not have been captured in the initial search.

### Synthesis

The final synthesis was summarized the findings to emphasize the role and effectiveness of the CPOT in pain assessment for non-verbal patients in critical care settings. This synthesis was also provided recommendations for practice based on the evidence gathered.

### Overview of CPOT

#### Development and purpose

The Critical Care Pain Observation Tool (CPOT) was developed in response to the need for an effective pain assessment method for patients in the intensive care unit (ICU) who are unable to verbally communicate their pain. The CPOT was first introduced by Payen as a solution to the limitations of self-report tools and to address the challenges of assessing pain in non-communicative patients (11). Its development was based on the recognition that traditional pain assessment methods were inadequate for critically ill patients, who often have altered consciousness or are sedated (12). The CPOT was designed to provide a reliable and objective measure of pain through behavioral and physiological indicators, thus enhancing pain management in ICU settings (4).

The Critical Care Pain Observation Tool (CPOT) is a rigorously validated instrument designed to assess pain in non-communicative critically ill patients. Psychometric evaluations highlight its strong reliability and validity, with high inter-rater reliability (kappa coefficient of 0.80–0.90) and effective differentiation between pain and non-pain states (13, 14). Its construct validity is supported by its correlation with other pain assessment tools, and it exhibits good sensitivity and specificity for diverse patient populations (15). By focusing on observable behaviors rather than self-reported pain, the CPOT meets the complex needs of critically ill patients and ensures accurate pain assessment in critical care settings (7, 16).

### Components of CPOT

The CPOT evaluates pain through four distinct behavioral indicators: facial expression, body movements, muscle tension, and ventilator compliance (for intubated patients) or vocalization (for non-intubated patients). Each component is scored from 0 to 2, with the total score ranging from 0 to 8 (2). The following descriptions and Table 1 summarize the CPOT scoring system and its interpretation (17–19).

1. Facial Expression:

**Table 1:** The CPOT scoring system and interpretation for non-verbal patients in critical care (2).

Component	Description	Interpretation
Facial expression	Evaluates grimacing or frowning	0 = relaxed or neutral, 1 = tense, 2 = grimacing
Body movements	Assesses extent of restlessness or agitation	0 = absence of movement or normal position, 1 = protection, 2 = restlessness or agitation
Muscle tension	Observes signs of muscle rigidity or clenching	0 = relaxed, 1 = tense, rigid, 2 = very tense or rigid
Ventilator compliance	Measures synchronization with the ventilator	0 = Fully compliant, 1 = coughing but tolerating, 2 = fighting ventilator (Poorly compliant)

Facial expression is assessed by observing the presence and intensity of grimacing or other signs of discomfort on the patient's face. Facial expressions are a key indicator of pain, with evidence showing that grimacing is a reliable marker of pain severity. A score of 0 indicates no signs of pain(relaxed or neutral), while a score of 2 reflects severe facial grimacing (2, 12).

2. Body Movements:

Body movements are evaluated based on the extent of restlessness or agitation. Increased body movements are typically associated with higher pain levels, as restlessness can be a behavioral response to pain. Scores range from 0, indicating no movement or normal position, to 2, indicating constant, severe agitation or restless (2, 5).

3. Muscle Tension:

Muscle tension is observed by noting physical signs such as clenched fists or a rigid posture. Muscle tension is a significant indicator of pain, as increased rigidity can correlate with higher pain intensity. Scores range from 0, with no muscle tension(relaxed), to 2, with severe muscle rigidity or tension (2, 5).

4. Ventilator Compliance:

Ventilator compliance for intubated patients assesses how well the patient synchronizes with the ventilator. Poor compliance or irregular breathing patterns can be indicative of pain or discomfort. This component is crucial for intubated patients, with scores ranging from 0, indicating full compliance, to 2, indicating poor compliance (2, 19, 20).

### Interpretation of CPOT scores

Behavioral pain scores should be interpreted differently from the patient's self-report pain intensity scores. More specifically, behavioral scores based on the nurse's observations are associated with the behavioral dimension of pain, and the patient's self-report of pain intensity relates to the sensory dimension of pain (21). Therefore, it is important to know that behavioral pain scales only allow the detection of the presence vs. absence of pain. Generally, a COPT score 0–2 indicates no or minimal pain whereas a CPOT score higher than 2 strongly suggests the presence of pain (2).

### Clinical applications of CPOT

#### Effectiveness in pain assessment

The CPOT has demonstrated robust effectiveness in identifying pain among ICU patients, often outperforming other pain assessment tools in terms of accuracy and reliability (22). Research indicates that the CPOT is highly effective in detecting pain in non-communicative patients, with studies showing that it consistently identifies pain levels with high sensitivity and specificity (23). Compared to other tools such as the Behavioral Pain Scale (BPS) and the FLACC scale, the CPOT has shown superior performance in distinguishing pain from other sources of discomfort, which is crucial in the complex ICU environment (17). A study found that CPOT provides a more detailed and accurate assessment of pain compared to the BPS, particularly in patients with varying levels of sedation (6).

#### Implementation in different settings

CPOT has been widely implemented across various ICU settings, demonstrating its versatility and adaptability. In general ICU settings, it has been employed successfully to monitor pain in critically ill patients who are unable to communicate verbally, leading to improved pain management practices (5). Its implementation extends to specialized ICUs, such as cardiac and neurocritical care units, where it has been adapted to address specific patient needs and conditions (2). For example, in cardiac ICUs, CPOT has been used to assess pain in patients recovering from major surgeries, while in neurocritical care units, modifications have been made to better accommodate patients with severe neurological impairments (23). These adaptations highlight CPOT's flexibility and effectiveness in diverse clinical environments.

#### Training and utilization

The integration of CPOT into clinical practice involves structured training programs for healthcare providers to ensure accurate and consistent use of the tool (24). Training typically includes educational sessions and hands-on workshops to familiarize ICU staff with CPOT's components and scoring system (11, 25). The tool's implementation has been associated with significant improvements in pain management, as it enables clinicians to make more informed decisions regarding analgesic interventions (26). Evidence suggests that CPOT facilitates better pain control and enhances overall patient comfort by providing a reliable means of assessing pain in critically ill patients (6). The consistent use of CPOT has also been linked to improved patient outcomes and a reduction in pain-related complications, emphasizing its impact on enhancing care quality in the ICU (27).

### Challenges and limitations

#### Limitations of CPOT

Despite its effectiveness, the CPOT is not without limitations. One notable challenge is related to inter-rater reliability. Studies have shown that while CPOT is generally reliable, there can be variability in scoring between different raters, which may affect the consistency of pain assessments (28, 29). Additionally, the applicability of CPOT can be limited in certain patient populations. For instance, patients with severe neurological conditions or those under deep sedation may present challenges in interpreting some behavioral indicators, such as facial expressions and muscle tension, which may not accurately reflect their pain levels (30). Moreover, in patients with certain conditions, such as those with facial paralysis or neuromuscular disorders or patients under neuromuscular blocking agents, the facial expression component of CPOT may not be a reliable indicator of pain (31, 32). These limitations emphasize the need for ongoing validation and possible adjustments of the tool for specific patient groups.

#### Barriers to implementation

Implementing CPOT effectively can be hindered by several barriers. One significant challenge is resource constraints, which can limit the availability of training programs and the integration of CPOT into routine clinical practice (33, 34). In some healthcare settings, especially in low-resource environments, the lack of sufficient training and educational resources can impede the effective use of CPOT (35, 36). Additionally, resistance to adopting new tools can be another barrier; healthcare providers may be hesitant to change established pain assessment practices, especially if they are unfamiliar with CPOT or perceive it as cumbersome (37). This resistance can be mitigated through targeted education and demonstrating the tool's benefits, but overcoming initial reluctance can be a significant hurdle (38). Finally, the integration of CPOT into existing electronic health record systems and workflows may require additional adjustments and technical support, which can pose logistical challenges (39).

In conclusion, while the CPOT offers a valuable approach to pain assessment in the ICU, it is essential to address its limitations, such as variability in inter-rater reliability and challenges in certain patient populations. Additionally, overcoming barriers related to resource constraints, training, and resistance to new practices is crucial for the successful implementation and utilization of CPOT in clinical settings.

### Comparisons with other tools

#### Comparison with other pain assessment tools

The Critical Care Pain Observation Tool (CPOT) is frequently compared with other pain assessment tools used in critical care settings, such as the Behavioral Pain Scale (BPS) and the Faces, Legs, Activity, Cry, Consolability (FLACC) scale (40). The BPS, another commonly used tool in ICUs, evaluates pain based on facial expressions, upper limb movements, and compliance with the ventilator, offering a comprehensive assessment of pain through observable behaviors (1). Research has demonstrated that CPOT and BPS are both effective in assessing pain in non-communicative patients, but CPOT generally offers a more comprehensive assessment by including muscle tension as an additional indicator (10, 22).

The FLACC scale, designed primarily for pediatric patients but also used in some adult settings, assesses pain based on facial expression, leg movement, activity, cry, and consolability (41). While the FLACC scale is beneficial for its simplicity and ease of use, CPOT is often preferred in adult critical care environments due to its more nuanced approach, particularly its focus on muscle tension and ventilator compliance, which are critical for assessing pain in intubated patients (42). Studies have indicated that CPOT may offer better sensitivity and specificity in identifying pain in patients who are deeply sedated or mechanically ventilated, where the FLACC scale might fall short (7, 43).

### Advantages and disadvantages

CPOT has several advantages over other tools like BPS and FLACC. One key advantage is its inclusion of muscle tension and ventilator compliance as indicators, which can provide a more detailed picture of pain, especially in mechanically ventilated patients (44). This additional detail can enhance pain assessment accuracy in critically ill patients whose pain responses may be less overt or complicated by their medical conditions (45). Additionally, CPOT's focus on observable behaviors and physiological responses helps mitigate the challenges associated with self-reporting tools and provides a reliable method for pain assessment in patients who cannot communicate (24).

However, CPOT is not without its disadvantages. One limitation is that it requires thorough training to ensure accurate and consistent application, which can be resource-intensive (35). Furthermore, while CPOT addresses some of the limitations of the BPS and FLACC, it may still face challenges in certain patient populations, such as those with severe neurological impairments where facial expressions or muscle tension might not accurately reflect pain (40). Additionally, inter-rater variability can be a concern, as with other observational tools, potentially affecting the reliability of pain assessments (46).

In summary, CPOT offers a more comprehensive approach to pain assessment in critical care compared to the BPS and FLACC scale, particularly through its inclusion of muscle tension and ventilator compliance. However, it also has limitations related to training requirements and applicability in certain patient populations, which need to be addressed to maximize its effectiveness in clinical practice.

### Potential innovations

There are several potential innovations that could enhance the CPOT and pain assessment practices in critical care. One promising area is the integration of technology to improve the accuracy and ease of CPOT administration. For instance, developing electronic versions of CPOT that incorporate automated scoring and real-time data analysis could reduce the burden on healthcare providers and minimize inter-rater variability (47). Studies have shown that electronic tracking can provide more accurate and timely data, reduce reliance on memory, and minimize biases associated with recalling past events, making it a more reliable option than paper-based records (47). This innovation enhances efficiency and aligns with the growing trend of digital transformation in healthcare, including critical care (48).

Advances in artificial intelligence(AI) and machine learning could further refine CPOT by analyzing patterns in pain indicators, enabling more precise pain assessments and predictive analytics (49). Research indicates that AI algorithms can identify subtle changes in patient data that human observers might miss, potentially leading to more timely interventions (50). By integrating these technologies, healthcare providers could enhance pain management protocols and improve patient outcomes.

Another innovation could involve expanding CPOT's behavioral indicators to include additional physiological indicators, such as blood pressure, heart rate and respiratory rate variability or biomarkers, which may offer a more comprehensive assessment of pain (51). While vital sign values generally increase during painful procedures, their effectiveness in the pain assessment remains questionable (52, 53). However, an integrated review study indicated that incorporating a wider range of physiological indicators could improve the reliability of pain evaluation, fostering a more holistic approach to patient care (54).

Furthermore, incorporating patient feedback mechanisms, where feasible, could improve the tool's responsiveness and accuracy by aligning it more closely with patient experiences and reported pain levels (55). Lastly, enhancing training programs with simulation-based learning and ongoing support could improve the consistency and reliability of CPOT use among clinicians (56).

### Limitation of the study

Despite the review's thorough evaluation, several limitations exist. The reliance on existing literature may overlook details in CPOT's use across specialized or diverse patient populations, such as those with severe neurological impairments or in pediatric and geriatric ICUs. Variability in training and implementation practices could affect CPOT's consistency and effectiveness. Additionally, the review does not include firsthand user experiences, potentially missing practical challenges.

## Discussion

One study compared the sensitivity of CPOT and BPS in mechanically ventilated patients showed that CPOT is more sensitive (76.5%) than BPS (62.7%). However, Both the CPOT and the BPS demonstrated strong criterion and discriminant validity (57). Additionally, a study on the validity and reliability of the CPOT revealed that its inter-rater reliability (IRR) ranged from fair to almost perfect, indicating that while the instrument's reliability is acceptable, it may vary. Conversely, the discriminant validity (DV) demonstrated a significant difference in mean scores between noxious (painful) and non-noxious (non-painful) procedures (20).

In other studies, the CPOT demonstrated moderate to high inter-rater reliability, as assessed by the Kappa coefficient from two or more raters. The values ranged from 0.79 to 0.94 across three studies (40, 58, 59). This strong agreement among assessors emphasizes CPOT's reliability as an observational tool in critical care settings. The authors noted that the tool's design allows for nuanced assessment through multiple indicators, including facial expressions, body movements, and muscle tension, and either compliance with ventilator (for intubated patients) or vocalization (for non-intubated patients which contribute to its superior performance in pain identification compared to traditional scales.

The Critical Pain Observation Tool (CPOT) provides a more comprehensive approach to pain assessment in critical care settings compared to the Behavioral Pain Scale (BPS) and the FLACC scale. One of the key advantages of CPOT is its incorporation of specific indicators such as muscle tension and ventilator compliance, which are particularly relevant for critically ill patients. By assessing muscle tension, CPOT captures a crucial aspect of pain expression that may not be fully represented in other scales. Additionally, ventilator compliance reflects the patient's ability to respond to pain stimuli while on mechanical ventilation, allowing for a more nuanced understanding of their pain experience. This multidimensional approach enhances the accuracy and effectiveness of pain assessment, ultimately leading to better pain management strategies for non-communicative patients in the ICU (2, 60, 61).

The CPOT has demonstrated considerable versatility and applicability across various ICU settings, specialized units such as cardiac and neurocritical care. In neurocritical care units, for instance, modifications to the tool have been implemented to better accommodate patients with severe neurological impairments. This adaptability underscores CPOT's potential to enhance pain assessment and management in diverse clinical contexts. By tailoring the tool to meet the specific needs of vulnerable populations, CPOT ensures that even those patients who are least able to communicate their pain receive the appropriate care they require. This capacity for customization not only improves pain management outcomes but also contributes to a more patient-centered approach in critical care settings (2, 5, 23).

## Conclusion

This review has highlighted the Critical Care Pain Observation Tool (CPOT) as a valuable instrument for pain assessment in critical care settings. The CPOT has demonstrated robust effectiveness in identifying pain among non-communicative ICU patients, often surpassing other tools such as the Behavioral Pain Scale (BPS) and the FLACC scale in terms of accuracy and reliability. Its comprehensive approach, which includes facial expression, body movements, muscle tension, and ventilator compliance, provides a nuanced and reliable measure of pain, especially in mechanically ventilated and deeply sedated patients. Despite its strengths, the CPOT faces limitations such as inter-rater variability and challenges in specific patient populations, which necessitate further refinement and research.

### Clinical implications

The clinical implications of using CPOT in the ICU are significant. The tool facilitates more accurate and objective pain assessment, which is crucial for effective pain management in critically ill patients who are unable to communicate their pain verbally. By improving pain detection and management, CPOT enhances patient comfort, potentially reduces pain-related complications, and contributes to overall better patient outcomes. The integration of CPOT into routine practice supports a more systematic approach to pain management, ensuring that pain is adequately assessed and addressed in a vulnerable patient population.

### Integration of CPOT into routine practice

Integrating the Critical Care Pain Observation Tool (CPOT) into routine practice in the ICU is essential for optimizing pain management in non-verbal patients. This process requires comprehensive training for ICU staff to ensure familiarity with CPOT's components and scoring system. Developing standardized protocols promotes consistent use, while engaging a multidisciplinary team fosters collaboration in pain assessment. Incorporating CPOT into electronic health records facilitates documentation and real-time monitoring of pain trends. Regular audits and feedback encourage adherence and highlight the tool's impact on care. Involving patients’ families in discussions about pain management provides valuable insights, and a framework for continuous quality improvement ensures ongoing evaluation and adaptation of the tool based on staff and patient feedback.

### Recommendations

To optimize the use of the Critical Care Pain Observation Tool (CPOT) in critical care settings, it is crucial to continue research aimed at enhancing its sensitivity and specificity, particularly for diverse patient populations such as those with severe neurological impairments or deep sedation. Future studies should focus on refining CPOT's indicators and evaluating its effectiveness in specialized ICUs like pediatric or geriatric units. Additionally, exploring technological advancements, such as electronic versions with automated scoring, could reduce inter-rater variability. Comprehensive training programs incorporating simulation-based learning are essential to ensure consistent application and improve clinician proficiency.
